# Supplementary material for: Longitudinal associations of epigenetic aging with cognitive aging in Hispanic/Latino adults from the Hispanic Community Health Study/Study of Latinos
Source: Aging (Albany NY). 2025 Sep 10;17(10):2455–74. doi: 10.18632/aging.206317 (PMC12606969; doi:10.18632/aging.206317)
Supplement: Supplementary Tables [file aging-17-10-206317-s001.pdf]

SUPPLEMENTARY TABLES

Supplementary Table 1. Correlations between chronological age and DNA methylation age estimated from 5 epigenetic clocks at each visit.

	Hannum	Horvath	PhenoAge	GrimAge	DunedinPACE*
Visit 1	0.80	0.78	0.78	0.86	0.80 (0.05)
Visit 2	0.78	0.76	0.76	0.86	0.78 (0.13)

P-value < 0.0001 for all correlations; *rescaled values (original scale shown in parentheses).

Supplementary Table 2. Correlation between epigenetic age acceleration within visit and across visits.

	Hannum	Horvath	PhenoAge	GrimAge	DunedinPACE
Hannum	0.89	0.88	0.68	0.34	0.21
Horvath	0.88	0.93	0.60	0.33	0.18
PhenoAge	0.70	0.64	0.81	0.50	0.45
GrimAge	0.41	0.38	0.57	0.91	0.53
DunedinPACE	0.27	0.21	0.48	0.53	0.81

P-value < 0.0001 for all correlations; Pairwise correlations between clocks at visit 2 are shown in white. Pairwise correlations between clocks across visits are shown in black. Pairwise correlations between clocks at visit 1 are shown in grey.

Supplementary Table 3. Cross-sectional associations of epigenetic clocks with individual cognitive test scores at each visit.

3A. B-SEVLT sum

		Model 1				Model 2				Model 3			
	EAA Measure	beta	SE	*P*-value	Adj_ *P*-value	beta	SE	*P*-value	Adj_ *P*-value	beta	SE	*P*-value	Adj_ *P*-value
Visit 1	Hannum	−0.005	0.004	0.259	1.00	−0.003	0.004	0.429	1.00	−0.003	0.004	0.427	1.00
	Horvath	−0.007	0.004	0.089	0.445	−0.005	0.004	0.155	0.775	−0.005	0.004	0.180	0.900
	PhenoAge	−0.007	0.003	**0.028**	0.140	−0.005	0.003	0.132	0.660	−0.004	0.003	0.160	0.800
	GrimAge	−0.016	0.005	**0.002**	**0.010**	−0.011	0.005	**0.028**	0.140	−0.009	0.005	0.068	0.340
	DunedinPACE*	−0.010	0.002	**5.4×10^{-5}**	**2.7×10^{-4}**	−0.007	0.002	**0.002**	**0.010**	−0.006	0.002	**0.009**	**0.045**
Visit 2	Hannum	−0.012	0.005	**0.008**	**0.040**	−0.010	0.004	**0.019**	0.095	−0.009	0.004	**0.036**	0.180
	Horvath	−0.012	0.005	**0.011**	0.055	−0.010	0.004	**0.021**	0.105	−0.009	0.004	**0.035**	0.175
	PhenoAge	−0.016	0.003	**6.3×10^{-6}**	**3.2×10^{-5}**	−0.015	0.003	**2.5×10^{-5}**	**1.2×10^{-4}**	−0.013	0.003	**0.0001**	**0.0005**
	GrimAge	−0.034	0.006	**3.0×10^{-8}**	**1.5×10^{-7}**	−0.030	0.006	**8.8×10^{-7}**	**4.4×10^{-6}**	−0.026	0.006	**2.5×10^{-5}**	**1.2×10^{-4}**
	DunedinPACE*	−0.014	0.003	**6.8×10^{-8}**	**3.4×10^{-8}**	−0.011	0.003	**7.4×10^{-6}**	**3.7×10^{-5}**	−0.009	0.003	**0.0003**	**0.0015**

3B. B–SEVLT recall

		Model 1				Model 2				Model 3			
	EAA Measure	beta	SE	*P*-value	Adj_ *P*-value	beta	SE	*P*–value	Adj_ *P*-value	beta	SE	*P*-value	Adj_ *P*-value
Visit 1	Hannum	−0.006	0.004	0.131	0.655	−0.005	0.004	0.2121	1.00	−0.005	0.004	0.252	1.00
	Horvath	−0.006	0.004	0.113	0.565	−0.005	0.004	0.1763	0.881	−0.005	0.004	0.249	1.00
	PhenoAge	−0.007	0.003	**0.026**	0.130	−0.005	0.003	0.093	0.465	−0.004	0.003	0.174	0.870
	GrimAge	−0.011	0.005	**0.023**	0.115	−0.008	0.005	0.125	0.625	−0.004	0.005	0.380	1.00

	DunedinPACE*	−0.008	0.002	**0.0004**	**0.002**	−0.006	0.002	**0.006**	**0.03**	−0.005	0.002	**0.044**	0.220
	Hannum	−0.006	0.005	0.231	1.00	−0.004	0.005	0.348	1.00	−0.004	0.005	0.433	1.00
	Horvath	−0.004	0.005	0.374	1.00	−0.003	0.005	0.505	1.00	−0.003	0.005	0.563	1.00
Visit 2	PhenoAge	−0.012	0.004	**0.0008**	**0.004**	−0.011	0.003	**0.002**	**0.010**	−0.010	0.004	**0.005**	**0.025**
	GrimAge	−0.020	0.006	**0.0008**	**0.004**	−0.017	0.006	**0.004**	**0.020**	−0.015	0.006	**0.016**	0.080
	DunedinPACE*	−0.008	0.003	**0.003**	**0.015**	−0.006	0.003	**0.029**	0.145	−0.004	0.003	0.106	0.530

3C. WF

		Model 1				Model 2				Model 3			
	EAA Measure	beta	SE	*P*-value	Adj_ *P*-value	beta	SE	*P*-value	Adj_ *P*-value	beta	SE	*P*-value	Adj_ *P*-value
	Hannum	−0.009	0.004	**0.029**	0.145	−0.007	0.004	0.075	0.375	−0.006	0.004	0.106	0.530
	Horvath	−0.011	0.004	**0.012**	0.060	−0.008	0.004	**0.027**	0.135	−0.008	0.004	**0.043**	0.215
Visit 1	PhenoAge	−0.014	0.003	**3.3×10^{-5}**	**1.7×10^{-4}**	−0.010	0.003	**0.001**	**0.005**	−0.009	0.003	**0.003**	**0.015**
	GrimAge	−0.024	0.005	**5.8×10^{-6}**	**2.9×10^{-5}**	−0.017	0.005	**0.0005**	**0.0025**	−0.016	0.005	**0.002**	**0.010**
	DunedinPACE*	−0.015	0.002	**1.2×10^{-9}**	**6.0×10^{-9}**	−0.011	0.002	**8.7×10^{-7}**	**4.4×10^{-4}**	−0.011	0.002	**1.3×10^{-5}**	**6.5×10^{-5}**
	Hannum	−0.015	0.005	**0.002**	**0.010**	−0.012	0.004	**0.007**	**0.035**	−0.011	0.004	**0.014**	0.070
	Horvath	−0.015	0.005	**0.001**	**0.005**	−0.013	0.004	**0.003**	**0.015**	−0.012	0.004	**0.005**	**0.025**
Visit 2	PhenoAge	−0.016	0.004	**1.1×10^{-5}**	**5.5×10^{-5}**	−0.014	0.003	**8.5×10^{-5}**	**4.3×10^{-4}**	−0.012	0.003	**0.0003**	**0.0015**
	GrimAge	−0.033	0.006	**1.4×10^{-7}**	**7.0×10^{-7}**	−0.025	0.006	**6.3×10^{-6}**	**3.2×10^{-5}**	−0.023	0.006	**0.0001**	**0.0005**
	DunedinPACE*	−0.016	0.003	**4.3×10^{-9}**	**2.2×10^{-8}**	−0.011	0.002	**3.2×10^{-6}**	**1.6×10^{-5}**	−0.010	0.003	**0.0001**	**0.0005**

3D. DSST

		Model 1				Model 2				Model 3			
	EAA Measure	beta	SE	*P*-value	Adj_ *P*-value	beta	SE	*P*-value	Adj_ *P*-value	beta	SE	*P*-value	Adj_ *P*-value
	Hannum	−0.009	0.004	**0.016**	0.080	−0.007	0.003	**0.032**	0.160	−0.007	0.003	**0.031**	0.155
	Horvath	−0.011	0.004	**0.005**	**0.025**	−0.008	0.003	**0.010**	0.050	−0.009	0.003	**0.008**	**0.040**
Visit 1	PhenoAge	−0.010	0.003	**0.001**	**0.005**	−0.006	0.003	**0.016**	0.080	−0.006	0.003	**0.019**	0.095
	GrimAge	−0.012	0.005	**0.013**	0.065	−0.008	0.004	**0.060**	0.300	−0.008	0.004	**0.053**	0.265
	DunedinPACE*	−0.010	0.002	**2.8×10^{-5}**	**1.4×10^{-4}**	−0.006	0.002	**0.003**	**0.015**	−0.006	0.002	**0.002**	**0.010**
	Hannum	−0.012	0.004	**0.003**	**0.015**	−0.010	0.004	**0.006**	**0.030**	−0.009	0.004	**0.011**	0.055
	Horvath	−0.013	0.004	**0.003**	**0.015**	−0.010	0.004	**0.005**	**0.025**	−0.009	0.004	**0.008**	**0.040**
Visit 2	PhenoAge	-0.012	0.003	**0.0001**	**0.0005**	−0.011	0.003	**1.1×10^{-4}**	**5.5×10^{-4}**	−0.010	0.003	**0.0004**	**0.002**
	GrimAge	−0.027	0.005	**5.9×10^{-7}**	**3.0×10^{-6}**	−0.023	0.005	**3.8×10^{-7}**	**1.9×10^{-6}**	−0.022	0.005	**2.4×10^{-6}**	**1.2×10^{-5}**
	DunedinPACE*	−0.012	0.002	**1.4×10^{-7}**	**7.0×10^{-6}**	−0.009	0.002	**2.0×10^{-5}**	**1.0×10^{-4}**	−0.007	0.002	**0.0002**	**0.001**

Model 1: Adjusted for age, gender, center, and Hispanic background; Model 2: Adjusted for variables in Model 1 + years of education, language preference; Model 3: Adjusted for variables in Model 2 + cardiovascular health score (Life's Simple 7 category); *DunedinPACE was rescaled to allow for comparison with other clocks (*P*-value shown for association on the original scale)

Supplementary Table 4. Association of change in global cognitive function with epigenetic age acceleration (EAA) at visit 1 for 5 clocks.

	Model 1				Model 2				Model 3			
EAA Measure	beta	SE	*P*-value	Adj_ *P*-value	beta	SE	*P*-value	Adj_ *P*-value	beta	SE	*P*-value	Adj_ *P*-value
V1 Hannum	−0.014	0.006	**0.0177**	0.088	−0.013	0.006	**0.026**	0.130	−0.012	0.006	**0.032**	0.160
V1 Horvath	−0.010	0.006	0.076	0.380	−0.009	0.006	0.106	0.530	−0.009	0.006	0.112	0.560
V1 PhenoAge	−0.015	0.005	**0.0016**	**0.008**	−0.013	0.005	**0.0040**	**0.020**	−0.012	0.005	**0.008**	**0.040**
V1 GrimAge	−0.027	0.007	**0.0002**	**0.001**	−0.026	0.007	**0.0005**	**0.0025**	−0.024	0.008	**0.002**	**0.010**
V1 DunedinPACE*	−0.009	0.003	**0.0028**	**0.014**	−0.008	0.003	**0.0082**	**0.041**	−0.007	0.003	**0.035**	0.175
V2 Hannum	−0.010	0.005	0.0653	0.362	−0.009	0.005	0.0853	0.426	−0.009	0.005	0.115	0.575
V2 Horvath	−0.010	0.005	0.0639	0.319	−0.009	0.005	0.0861	0.430	−0.009	0.005	0.096	0.480
V2 PhenoAge	−0.016	0.004	**9.7×10^{-5}**	**4.8×10^{-4}**	−0.016	0.004	**0.0002**	**0.001**	−0.015	0.004	**0.0005**	**0.0025**
V2 GrimAge	−0.032	0.007	**9.2×10^{-6}**	**4.6×10^{-5}**	−0.031	0.007	**1.7×10^{-5}**	**8.5×10^{-5}**	−0.029	0.007	**0.0001**	**0.0005**
V2 DunedinPACE*	−0.009	0.003	**0.0006**	**0.003**	−0.009	0.003	**0.0021**	**0.010**	−0.007	0.003	**0.0112**	0.056

Model 1: Adjusted for age, gender, center, and Hispanic background; Model 2: Adjusted for variables in Model 1 + years of education, language preference; Model 3: Adjusted for variables in Model 2 + cardiovascular health (Life's Simple 7 category); *DunedinPACE was rescaled to allow for comparison with other clocks (*P*-value shown for association on the original scale).

Supplementary Table 5. Association of change in individual cognitive tests scores with change (Δ) in epigenetic age acceleration (EAA) between visit 1 and visit 2 for 5 clocks.

5A. B-SEVLT sum change

EAA Measure	Model 1				Model 2				Model 3			
	Beta	SE	*P*-value	Adj_ *P*-value	Beta	SE	*P*-value	Adj_ *P*-value	Beta	SE	*P*-value	Adj_ *P*-value
Δ Hannum	−0.028	0.061	0.642	1.00	−0.025	0.060	0.681	1.00	−0.015	0.061	0.811	1.00
Δ Horvath	−0.090	0.076	0.240	1.00	−0.090	0.076	0.237	1.00	−0.079	0.076	0.301	1.00
Δ PhenoAge	−0.075	0.038	**0.048**	0.24	−0.078	0.038	**0.039**	0.19	−0.069	0.038	0.070	0.35
Δ GrimAge	−0.078	0.087	0.371	1.00	−0.089	0.087	0.309	1.00	−0.073	0.088	0.407	1.00
Δ DunedinPACE*	−0.021	0.028	0.461	1.00	−0.018	0.028	0.519	1.00	−0.011	0.028	0.685	1.00

5B. B-SEVLT recall change

EAA Measure	Model 1				Model 2				Model 3			
	Beta	SE	*P*-value	Adj_ *P*-value	Beta	SE	*P*-value	Adj_ *P*-value	Beta	SE	*P*-value	Adj_ *P*-value
Δ Hannum	0.055	0.061	0.370	1.00	0.057	0.061	0.348	1.00	0.059	0.061	0.339	1.00
Δ Horvath	0.002	0.077	0.979	1.00	0.001	0.077	0.987	1.00	−0.002	0.077	0.980	1.00
Δ PhenoAge	−0.047	0.038	0.219	1.00	−0.050	0.038	0.186	0.93	−0.049	0.038	0.202	1.00
Δ GrimAge	−0.089	0.089	0.314	1.00	−0.100	0.088	0.258	1.00	−0.093	0.089	0.293	1.00
Δ DunedinPACE*	−0.010	0.028	0.722	1.00	−0.008	0.028	0.783	1.00	−0.005	0.029	0.857	1.00

5C. WF change

EAA Measure	Model 1				Model 2				Model 3			
	Beta	SE	*P*-value	Adj_ *P*-value	Beta	SE	*P*-value	Adj_ *P*-value	Beta	SE	*P*-value	Adj_ *P*-value
Δ Hannum	0.030	0.061	0.628	1.00	0.028	0.061	0.647	1.00	0.039	0.061	0.5198	1.00
Δ Horvath	0.005	0.077	0.950	1.00	−0.002	0.076	0.980	1.00	0.007	0.077	0.9315	1.00

Δ PhenoAge	−0.037	0.038	0.331	1.00	−0.044	0.038	0.251	1.00	−0.037	0.038	0.3284	1.00
Δ GrimAge	−0.216	0.089	**0.015**	0.075	−0.234	0.088	**0.008**	**0.04**	−0.221	0.088	**0.013**	0.065
Δ DunedinPACE*	−0.049	0.028	0.080	0.40	−0.049	0.028	0.078	0.39	−0.048	0.028	0.089	0.445

5D. DSST change

	Model 1				Model 2				Model 3			
**EAA Measure**	**Beta**	**SE**	***P*-value**	**Adj_ *P*-value**	**Beta**	**SE**	***P*-value**	**Adj_ *P*-value**	**Beta**	**SE**	***P*-value**	**Adj_ *P*-value**
Δ Hannum	−0.085	0.062	0.171	0.855	−0.090	0.062	0.147	0.735	−0.073	0.063	0.243	1.00
Δ Horvath	−0.098	0.078	0.208	1.00	−0.105	0.078	0.178	0.890	−0.091	0.078	0.246	1.00
Δ PhenoAge	−0.134	0.039	**0.0006**	**0.003**	−0.139	0.039	**0.0004**	**0.002**	−0.125	0.039	**0.0015**	**0.0075**
Δ GrimAge	−0.319	0.090	**0.0004**	**0.002**	−0.334	0.090	**0.0002**	**0.001**	−0.313	0.090	**0.0005**	**0.0025**
Δ DunedinPACE*	−0.058	0.029	**0.042**	0.210	−0.059	0.029	**0.040**	0.200	−0.051	0.029	0.079	0.395

Model 1: Adjusted for V1 age acceleration, age, gender, center, and Hispanic background; Model 2: Adjusted for variables in Model 1 + years of education, language preference; Model 3: Adjusted for variables in Model 2 + cardiovascular health (Life's Simple 7 category); *DunedinPACE was rescaled to allow for comparison with other clocks.

Supplementary Table 6. Comparison of the magnitude of associations between change in DSST and epigenetic aging with the magnitude of association between change in DSST and APOE4.

	beta	SE	*P*-value
APOE4 alleles dosage	−0.084	0.042	**0.043**
V1 PhenoAge	−0.010	0.004	**0.019**
V1 GrimAge	−0.028	0.007	**<0.0001**
V1 DunedinPACE*	−0.010	0.003	**0.002**
Δ PhenoAge	−0.134	0.039	**0.0006**
Δ GrimAge	−0.319	0.090	**0.0004**
Δ DunedinPACE*	−0.058	0.029	**0.042**

*Model adjusted for age, gender, center, and Hispanic background.

Supplementary Table 7. Association of cognitive aging measures with estimates of GrimAge acceleration derived from multiple algorithms.

Cognitive aging measure	EAA Measure	Beta/Odds Ratio	SE/95% CI	*P*-value
V1 Global Cognitive Function	V1 PC GrimAge*	−0.018	0.004	**9.1×10^{-6}**
	V1 GrimAge	−0.012	0.003	**8.2×10^{-5}**
	V1 GrimAge2	−0.012	0.003	**9.9×10^{-5}**
V2 Global Cognitive Function	V2 PC GrimAge*	−0.032	0.005	**3.3×10^{-10}**
	V2 GrimAge	−0.020	0.004	**8.4×10^{-8}**
	V2 GrimAge2	−0.022	0.003	**8.5×10^{-11}**
MCI at V2	V1 PC GrimAge*	1.07	1.04; 1.11	**5.4×10^{-5}**
	V1 GrimAge	1.05	1.02; 1.07	**6.6×10^{-4}**
	V1 GrimAge2	1.05	1.03; 1.08	**2.5×10^{-5}**
	V2 PC GrimAge*	1.09	1.05; 1.12	**1.4×10^{-6}**
	V2 GrimAge	1.06	1.04; 1.09	**1.3×10^{-6}**
	V2 GrimAge2	1.06	1.04; 1.09	**2.3×10^{-7}**
Significant cognitive decline at V2	V1 PC GrimAge*	1.05	1.02; 1.08	**1.6×10^{-3}**
	V1 GrimAge	1.02	1.00; 1.05	**2.9×10^{-2}**
	V1 GrimAge2	1.03	1.01; 1.05	**4.5×10^{-3}**
	V2 PC GrimAge*	1.07	1.04; 1.10	**7.1×10^{-6}**
	V2 GrimAge	1.05	1.03; 1.07	**2.0×10^{-5}**
	V2 GrimAge2	1.05	1.03; 1.07	**8.4×10^{-7}**

*Data shown in manuscript's Tables. Models are adjusted for age, gender, center, and Hispanic background.

Supplementary Table 8. Association of DNA methylation components of GrimAge version 2 estimated at each visit with cognitive aging measures.

Visit 1	V1 Global Cognitive Function			MCI at V2			Significant cognitive decline at V2			Global Cognitive Function Change between V1 and V2		
	**Beta (SE)**	***P***	**Adj_*P***	**Beta (SE)**	***P***	**Adj_*P***	**Beta (SE)**	***P***	**Adj_*P***	**Beta (SE)**	***P***	**Adj_*P***
ADM	−0.005 (0.013)	0.69	1.00	0.165 (0.060)	**0.006**	0.06	0.118 (0.048)	**0.0138**	0.14	−0.058 (0.024)	**0.0147**	0.15
B2M	−0.011 (0.011)	0.33	1.00	0.106 (0.047)	**0.025**	0.25	0.047 (0.040)	0.24	1.00	−0.036 (0.020)	**0.0694**	0.69
Cystatin C	−0.027 (0.011)	**0.015**	0.15	0.115 ((0.048)	**0.017**	0.17	0.087 (0.040)	**0.0303**	0.30	−0.053 (0.020)	**0.0081**	0.08
GDF15	−0.015 (0.011)	0.16	1.00	0.165 (0.045)	**0.0003**	**0.003**	0.183 (0.042)	**1.4×10^{-5}**	**1.4×10^{-4}**	−0.070 (0.020)	**0.0004**	**0.004**
Leptin	−0.003 (0.017)	0.87	1.00	0.038 (0.075)	0.61	1.00	0.056 (0.062)	0.36	1.00	0.012 (0.031)	0.68	1.00
logA1C	−0.034 (0.011)	**0.002**	**0.02**	0.265 (0.048)	**3.3×10^{-8}**	**3.3×10^{-7}**	0.232 (0.041)	**1.2×10^{-8}**	**1.2×10^{-7}**	−0.106 (0.020)	**8.0×10^{-8}**	**8.0×10^{-7}**
logCRP	−0.025 (0.011)	**0.030**	0.30	0.141 (0.051)	**0.005**	0.05	0.160 (0.042)	**0.0001**	**0.001**	−0.084 (0.020)	**3.9×10^{-5}**	**3.9×10^{-4}**
Pack Yrs	−0.042 (0.012)	**0.0003**	**0.003**	0.167 (0.050)	**0.0007**	**0.007**	0.104 (0.042)	**0.0144**	0.14	−0.052 (0.021)	**0.0139**	0.14
PAI1	0.007 (0.012)	0.57	1.00	0.176 (0.052)	**0.0007**	**0.007**	0.198 (0.043)	**3.8×10^{-6}**	**3.8×10^{-5}**	−0.074 (0.021)	**0.0004**	**0.004**
TIMP1	−0.022 (0.011)	0.05	1.00	0.098 (0.050)	0.05	0.50	0.087 (0.041)	**0.0351**	0.35	−0.048 (0.020)	**0.0189**	0.19

Visit 2	V2 Global Cognitive Function			MCI at V2			Significant cognitive decline at V2			Global Cognitive Function Change between V1 and V2		
	**Beta (SE)**	***P***	**Adj_*P***	**Beta (SE)**	***P***	**Adj_*P***	**Beta (SE)**	***P***	**Adj_*P***	**Beta (SE)**	***P***	**Adj_*P***
ADM	−0.037 (0.017)	**0.0296**	0.30	0.126 (0.061)	**0.0394**	0.39	0.092 (0.049)	0.06	0.60	−0.052 (0.024)	**0.0341**	0.34
B2M	−0.028 (0.014)	**0.0414**	0.41	0.062 (0.047)	0.18	1.00	0.001 (0.040)	0.98	1.00	−0.010 (0.020)	0.61	1.00
Cystatin C	−0.042 (0.014)	**0.0023**	0.02	0.042 (0.049)	0.38	1.00	0.019 (0.040)	0.63	1.00	−0.016 (0.020)	0.40	1.00
GDF15	−0.046 (0.014)	**0.0008**	0.008	0.048 (0.046)	0.31	1.00	0.035 (0.039)	0.37	1.00	−0.029 (0.019)	0.13	1.00
Leptin	−0.019 (0.021)	0.36	1.00	0.027 (0.073)	0.71	1.00	0.053 (0.060)	0.38	1.00	−0.005 (0.030)	0.88	1.00
logA1C	−0.077 (0.014)	**3.0×10^{-8}**	**3.0×10^{-7}**	0.298 (0.047)	**3.1×10^{-10}**	**3.1×10^{-9}**	0.187 (0.040)	**3.6×10^{-6}**	**3.6×10^{-5}**	−0.103 (0.020)	**1.7×10^{-7}**	**1.7×10^{-6}**
logCRP	−0.062 (0.015)	**2.2×10^{-5}**	**2.2×10^{-4}**	0.173 (0.052)	**0.0008**	**0.008**	0.116 (0.042)	**0.0059**	0.06	−0.075 (0.021)	**0.0003**	**0.003**
Pack Yrs	−0.054 (0.015)	**0.0002**	**0.002**	0.128 (0.050)	**0.0106**	0.11	0.059 (0.042)	0.1594	1.00	−0.042 (0.021)	**0.0447**	0.45
PAI1	−0.038 (0.015)	**0.0117**	0.12	0.196 (0.052)	**0.0002**	**0.002**	0.126 (0.043)	**0.0032**	**0.032**	−0.075 (0.021)	**0.0004**	**0.004**
TIMP1	−0.031 (0.014)	**0.0265**	0.26	0.033 (0.049)	0.50	1.00	0.016 (0.040)	0.69	1.00	−0.027 (0.020)	0.17	1.00

Models are adjusted for age, gender, center, and Hispanic background. Adj_P: *P*-value adjusted for the number of surrogate biomarkers. ADM: Adrenomedullin; B2M: β2-microglobulin; GDF15: Growth differentiation factor 15; logA1c: hemoglobin A1C (log transformed); logCRP: C-reactive protein (log transformed); Pack Yrs: Smoking in pack/year; PAI-1: Plasminogen activator inhibitor 1; TIMP1: tissue inhibitor metalloproteinase 1
